# Immunohistochemical detection of mutations in the epidermal growth factor receptor gene in lung adenocarcinomas using mutation-specific antibodies

**DOI:** 10.1186/1746-1596-8-27

**Published:** 2013-02-18

**Authors:** Yan Xiong, Yun Bai, Nufatt Leong, Todd S Laughlin, Paul G Rothberg, Haodong Xu, Lin Nong, Jing Zhao, Ying Dong, Ting Li

**Affiliations:** 1 Department of Pathology, Peking University First Hospital, 7 Xishiku Street, Xicheng District, Beijing, 100034, China; 2 Department of Pathology and Laboratory Medicine, University of Rochester Medical Center, 601 Elmwood Avenue, Rochester, NY, 14642, USA

**Keywords:** Lung adenocarcinoma, Epidermal growth factor receptor, Mutation, Immunohistochemistry

## Abstract

**Background:**

The recent development of antibodies specific for the major hotspot mutations in the epidermal growth factor receptor (EGFR), L858R and E746_A750del, may provide an opportunity to use immunohistochemistry (IHC) as a screening test for *EGFR* gene mutations. This study was designed to optimize the IHC protocol and the criteria for interpretation of the results using DNA sequencing as the gold-standard.

**Methods:**

Tumor sections from fifty lung adenocarcinoma specimens from Chinese patients were immunostained using L858R and E746_A750del-specific antibodies using three different antigen retrieval solutions, and the results were evaluated using three different sets of criteria. The same specimens were used for DNA purification and analysis of EGFR gene mutations.

**Results:**

In this study the optimal buffer for antigen retrieval was EDTA (pH 8.0), and the optimal scoring method was to call positive results when there was moderate to strong staining of membrane and/or cytoplasm in >10% of the tumor cells. Using the optimized protocol, L858R-specific IHC showed a sensitivity of 81% and a specificity of 97%, and E746_A750del-specific IHC showed a sensitivity of 59% and a specificity of 100%, both compared with direct DNA analysis. Additionally, the mutant proteins as assessed by IHC showed a more homogeneous than heterogeneous pattern of expression.

**Conclusions:**

Our data demonstrate that mutation-specific IHC, using optimized procedures, is a reliable prescreening test for detecting EGFR mutations in lung adenocarcinoma.

**Virtual Slides:**

The virtual slide(s) for this article can be found here: http://www.diagnosticpathology.diagnomx.eu/vs/2059012601872392

## Background

Somatic mutations within the tyrosine kinase (TK) domain of the epidermal growth factor receptor (EGFR) gene are found in approximately 30% of lung adenocarcinomas in Asian populations [1]. Studies support that some of these activating mutations are not only reliable predictors of response to the small molecule EGFR tyrosine kinase inhibitors (TKIs) gefitinib and erlotinib but also prognostic factors for survival [2-4]. Among numerous TK domain mutations, 85–90% are exon 19 E746_A750 deletions or exon 21 L858R point mutations [5]. A variety of DNA-based molecular methods are used to detect EGFR mutations. These methods have respective advantages and disadvantages, with no consensus on which one is the best. For example, direct sequencing of PCR-amplified genomic DNA can detect all mutations in the regions analyzed, but has limited analytical sensitivity when the tumor cells are not a large fraction of the specimen. The amplification refractory mutation system (ARMS) assay is more sensitive, but detects fewer mutations, usually only one per reaction. In general, direct analysis of DNA is expensive because of the cost of the equipment and reagents. In addition it is technically complex, and usually done in laboratories that specialize in molecular pathology [6].

Yu et al. [7] developed mutation specific rabbit monoclonal antibodies against the two most common EGFR mutations and showed that these antibodies can be applied to the immunohistochemical (IHC) detection of these mutations in formalin-fixed paraffin-embedded (FFPE) tissue. Several independent groups have investigated the sensitivity and specificity of these antibodies in the detection of EGFR mutations in non-small cell lung cancer (NSCLC). Most of them confirmed a high degree of specificity, but the reported sensitivities were quite variable ranging from 24% to 100% (Table 1) [8-14]. This inconsistency may be related to differences in methodology and interpretation [10,13,15], as well as population specific differences in gene mutations and differences in the level of protein expression [16]. This inconsistency suggests that further study is needed in diverse populations before EGFR mutation-specific IHC can be implemented as a clinical tool.

**Table 1:** Literature review of sensitivity and specificity of mutation-specific immunohistochemistry

**Reference**	**N**	**L858R**		**E746_A750del**	
		**Sensitivity**	**Specificity**	**Sensitivity**	**Specificity**
Simonetti et al. [8]	78	100%	100%	63%	100%
Kato Y et al. [9]	70	75%	97%	82%	100%
Kitamura A et al. [10]	343	36%	97%	40%	99%
Brevet M et al. [11]	194	76%	100%	67%	100%
Wu SG et al. [12]	143	88	77%	94%	95%
Kozu Y et al. [13]	577	76%	98%	42%	99%
Hofman P et al. [14]	154	24%	98%	55%	97%

In the study reported here we optimized the methodology and interpretive aspects of IHC for detection of EGFR mutations, and evaluated the success of this effort by comparison with DNA sequencing. This study investigated the staining protocol, staining pattern, scoring methods, and cut off value to determine the diagnostic power of EGFR mutation-specific IHC in Chinese lung adenocarcinoma patients.

## Methods

### Patient samples

Samples for study were selected according to the following criteria: lung adenocarcinoma, surgically resected, primary, solitary and no preoperative therapy. A total of 50 cases were collected retrospectively and prospectively from the Department of Pathology, Peking University First Hospital during January 2010 to January 2012.

All specimens were dissected and immersed in 10% neutral buffered formalin, then fixed overnight. The number of sections for histology depended on the greatest dimension of tumors*, i.e*. one section per centimeter. If a tumor was less than 2 cm in greatest dimension, the tumor was totally sampled for microscopic examination. Sectioned tissues were embedded in paraffin routinely.

Informed consent for the use of these specimens for medical studies was obtained before surgery.

### Immunohistochemistry

50 tissue blocks were cut into 4-μm-thick whole sections. EGFR mutation specific antibodies were Rabbit XP® mAbs obtained from Cell Signaling Technology (Danvers, MA), 6B6 specific for the E746-A750del mutation, and 43B2 for the L858R mutation. The antibodies were diluted 1:100 with antigen retrieval buffer before use. The antigen retrieval buffers tested were sodium citrate (pH 6.0), EDTA (pH 8.0) and EDTA (pH 9.0). Cytokeratin AE1/AE3 IHC was used as a quality control for tissue and protocol. The IHC protocol is described in greater detail in the Additional file 1.

### IHC scoring

Three sets of criteria were used for interpretation of the IHC results, referred to as Score A, B and C, respectively, in this study. A positive result using score A was moderate to strong staining of membrane and/or cytoplasm in >10% tumor cells [15]. A positive result using score B was membrane staining in >10% tumor cells with any intensity [10]. A positive result using score C was membrane and/or cytoplasmic staining in >50% of the tumor cells with any intensity [13]. In this study all 50 specimens were analyzed using Score A, B and C separately, so as to evaluate the validity of these scoring methods by comparing to the results of DNA sequencing. Both the intensity and percentage of stained cells were assessed at low magnification (objective magnification ×10). The distribution of staining, membrane or cytoplasm, was assessed at high magnification (objective magnification ×40). Four experienced pathologists (Yan Xiong, Ying Dong, Lin Nong and Jing Zhao) reviewed all of the slides independently, and then replicated the analysis 16 to 18 weeks later. The intra- and inter-observer reliability was analyzed.

### DNA sequencing

#### DNA preparation

H&E stained sections of FFPE tissue were reviewed for each sample and those with greater than 50% tumor volume were selected for molecular testing. Genomic DNA was extracted using the QIAamp DNA FFPE Tissue Kit (Qiagen, Valencia, CA) according to the manufacturer’s protocol.

#### Mutant-enriched PCR for EGFR exon 19 and 21

All samples were studied by DNA sequencing after mutation enriched PCR of exons 19 and 21. The PCR was done in a total volume of 12 μL with primers at a final concentration of 1 μM each, 50 μM of each dNTP, 0.75 units of HotStar*Taq* DNA polymerase, and 1.2 μL of the 10X buffer provided by the enzyme manufacturer (Qiagen). Template was added in a volume of 1 μL containing approximately 50 ng of DNA. An oligonucleotide clamp was added to suppress amplification of the normal sequence and enhance amplification of the L858R mutation and the exon 19 deletions. The clamp was synthesized with several locked nucleic acid (LNA) positions to increase its avidity. The clamps for exons 19 (CLMP1) and 21 (CLMP2) were used at final concentrations of 25 nM, and 5 nM, respectively. The sequences of the primers and clamps are described in Table 2. The PCR primers were synthesized with M13 tail sequences appended to the 5′-end to facilitate sequencing. The reactions were cycled 40 times between 95°C for 10 seconds, 68°C for 15 seconds and 72°C for 20 seconds, preceded by 10 minutes at 95°C, and followed by 5 minutes at 72°C. The primers were purchased from Integrated DNA Technologies (Coralville, IA) and the LNA clamp was purchased from Biosynthesis, Inc. (Lewisville, TX).

**Table 2:** Sequences of oligodeoxyribonucleotides

**Name**	**Sequence (5′→3′)**^**1**^	**Position**
19 F	GCCAGTTAACGTCTTCCTTCTCTC	Intron 18
19R	YGAGCAGGGTCTAGAGCAGAGCA	Intron 19
21 F	CGGATGCAGAGCTTCTTCCCATG	Intron 20
21R	CTAGTGGGAAGGCAGCCTGGTC	Intron 21
CLMP1	+T+T+A+A+GA+GA+A+G+C+AA+C+A+T+CT-C3	Exon 19
CLMP2	+T+G+G+G+C+T+GGCA-C3	Exon 21

^1^Other than the bases A, T, C and G, the oligos are described by a Y for mixed T and C; the positions of the LNA nucleotides (+ before the LNA) in the clamps (CLMP1 and CLMP2); and the C3 spacer (-C3) on the 3′ terminus of the clamps.

#### DNA sequencing for EGFR exon 19 and 21

The amplicons were treated with ExoSap (Amersham Biosciences, Piscataway, NJ) to remove the primers and dNTPs; then 1 μL was sequenced using the M13 tail primers as sequencing primers and Applied Biosystems (ABI, Foster City, CA) BigDye Terminator v.3.1 chemistry. The sequencing reactions were purified using the CleanSeq system (Agencourt Bioscience, Beverly, MA) and then resolved by capillary electrophoresis on the ABI 3100 Prism Genetic Analyzer.

### Statistical analysis

Statistical analysis was done using the statistics software SPSS V16.0 (SPSS Inc., Chicago, IL). Fleiss’ Kappa was used to determine inter-observer agreement. Cohen’s Kappa was used to determine intra -observer agreement and agreement of IHC and DNA sequencing. A Kappa value between 0.81 and 1.0 was defined as nearly perfect agreement, between 0.61 and 0.8 as substantial agreement, between 0.41 and 0.60 as moderate agreement, between 0.21 and 0.40 as fair agreement, between 0.00 and 0.20 as slight agreement. For each Kappa, the 95% confidence interval (CI) was calculated. Difference was considered significant (P < 0.05), if the lower and upper boundary of the 95% CI showed no overlap.

### Ethical approval

All experiments above have been performed with the approval of Peking University First Hospital Ethics Committee.

## Results

### DNA sequencing

In the total cohort of 50 samples L858R was identified in 16 cases, a deletion in exon 19 in 17 cases, and neither of them in 17 cases. Of the 17 cases with exon 19 deletion, 14 had a p.E746_A750del (c.del2235_2249 on the DNA level), one had a p.L747_T751del (c.2240_2254del), one had a p.L747_P753delinsS (c.2240_2257del), and one had a p.L747_T751delinsPT (c.2239_2253delinsCCAACG) that had not been previously reported. In our study, of all 33 cases with EGFR mutations, L858R and E746_A750del together comprised 90% (30/33) and the others, including L747_T751del, L747_P753delinsS and L747_T751delinsPT, comprised 10%, which was concordant with other studies [17]. From this point of view L858R and E746_A750del are recognized as the most common mutations and the other mutation types are described as uncommon mutations.

### Evaluation of antigen retrieval buffer

**Figure 1:**
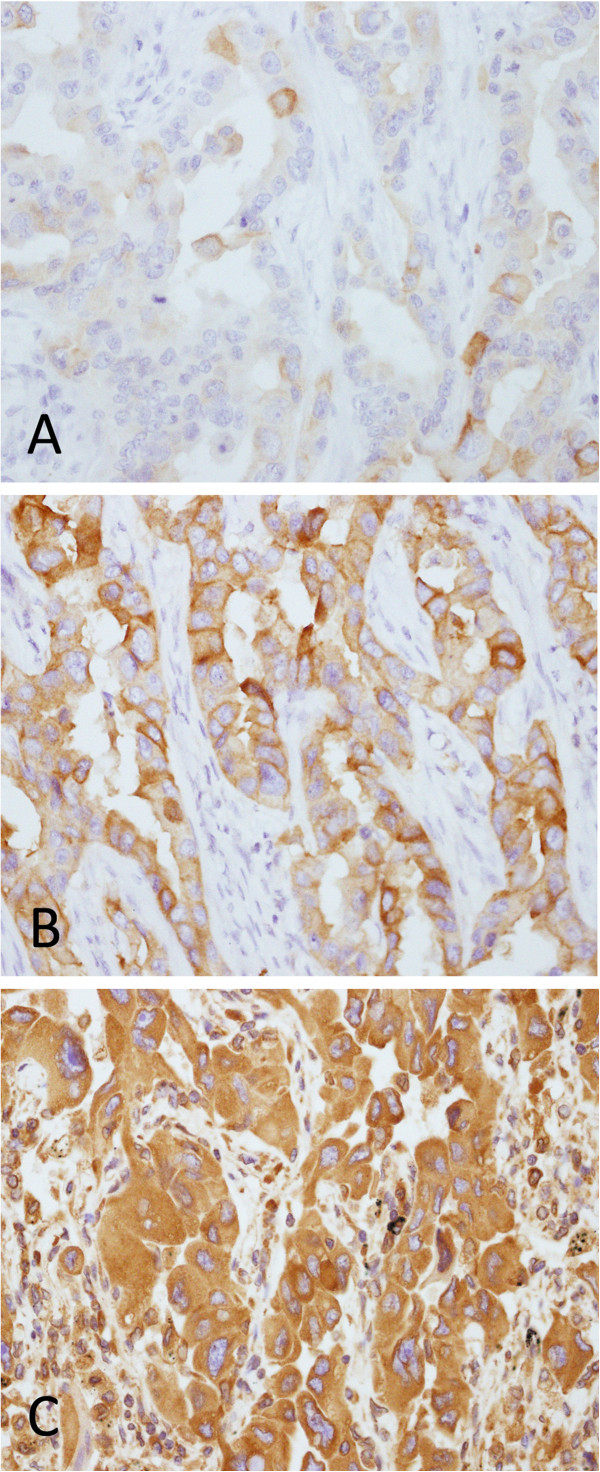
**Sample case of predominant solid adenocarcinoma immunostained with E746_A750del-specific antibody using different antigen retrieval buffers (original magnification x400). A** Sodium citrate (pH 6.0). **B** EDTA (pH 8.0). **C** EDTA (pH 9.0).

We evaluated three different antigen retrieval buffers on all 50 specimens to optimize the IHC results: sodium citrate (pH 6.0), EDTA (pH 8.0) and EDTA (pH 9.0). Slides in the EDTA (pH 8.0) group showed the best histological pictures with strongly specific staining and minimal background. The intensity of the positive cells in the sodium citrate (pH 6.0) group was too faint to distinguish from the background. Mesenchymal cells on slides exposed to EDTA (pH 9.0) were stained as strong as tumor cells, which made it impossible to identify the specificity of staining (Figure 1). Inter-observer agreement was nearly perfect in the EDTA (pH 8.0) group, substantial in the sodium citrate (pH 6.0) group and moderate in the EDTA (pH 9.0) group. The Fleiss’ Kappa (95% confidence interval) was 0.912 (0.862, 0.962), 0.753 (0.677, 0.829), and 0.643(0.558, 0.728) in the three groups, respectively. The difference between EDTA (pH 8.0) and the others was significant (P < 0.05). Intra-observer agreement was highest in EDTA (pH 8.0), moderate in sodium citrate (pH 6.0), and lowest in EDTA (pH 9.0). The Cohen’s Kappa (95% confidence interval) was 0.955 (0.918, 0.992), 0.853 (0.790, 0.916), and 0.801 (0.730, 0.872), respectively. The difference between EDTA (pH 8.0) and the others was statistically significant (P < 0.05) (Table 3).

**Table 3:** Intra- and inter-observer agreement based on slides treated with different antigen retrieval buffers

**Antigen retrieval buffer**	**Inter-observer agreement** **Fleiss’ Kappa**		**Intra-observer agreement** **Cohen’s Kappa**	
	**Value**	**95% CI**	**Value**	**95% CI**
**EDTA (pH 8.0)**	0.912	(0.862, 0.912)	0.955	(0.918, 0.992)
**Na Citrate (pH 6.0)**	0.753	(0.677, 0.829)	0.853	(0.790, 0.916)
**EDTA (pH 9.0)**	0.643	(0.558, 0.728)	0.801	(0.730, 0.872)
**P**	< 0.05		< 0.05	

### IHC results

**Figure 2:**
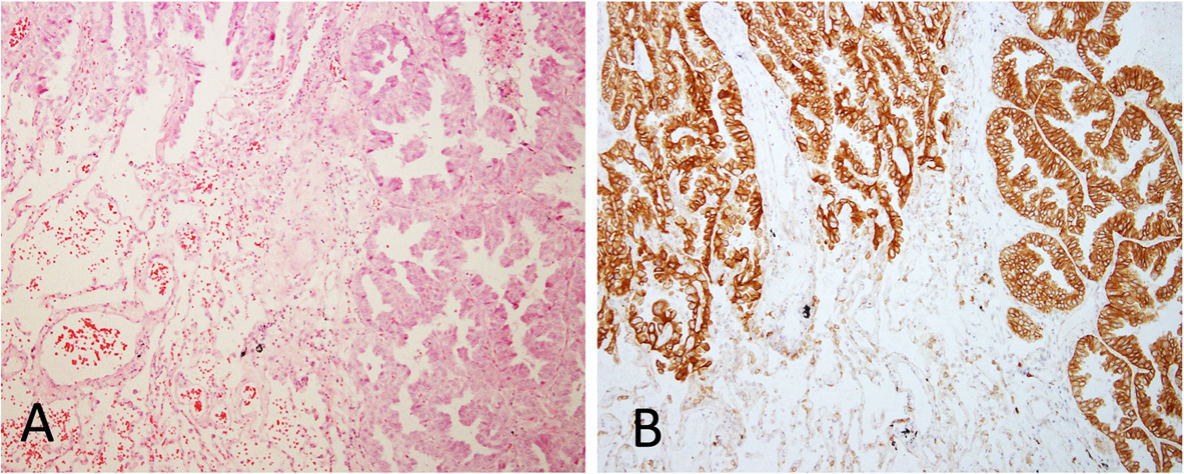
**Predominant acinar adenocarcinoma with adjacent normal alveoli. A** H & E (original magnification x100). **B** The cytoplasm and membrane of the tumor cells were stained strongly with L858-specific antibody. In contrast, the adjacent normal alveolar epithelial cells were completely negative (original magnification x100).

**Figure 3:**
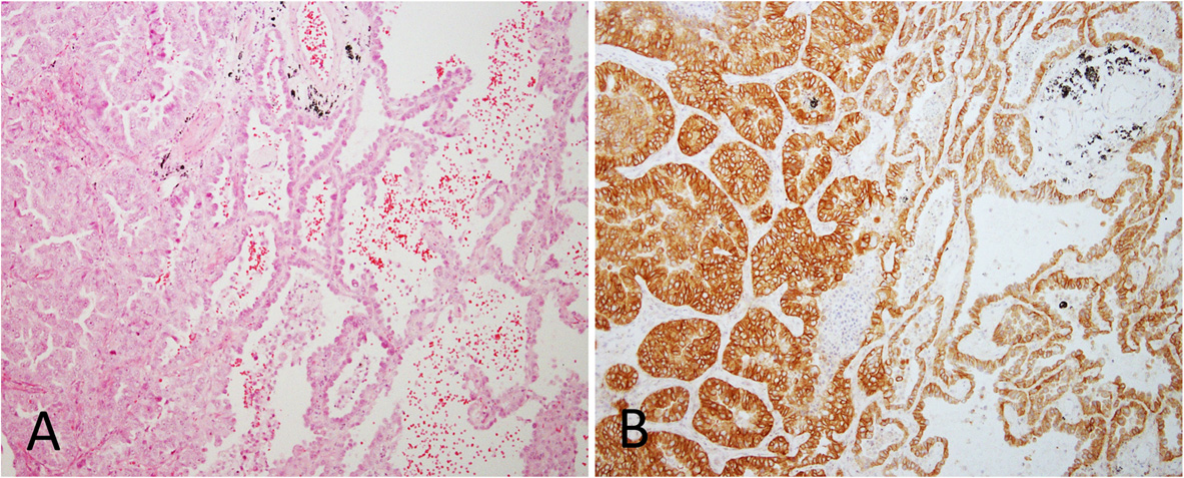
**Adenocarcinoma with acinar and lepidic patterns. A** H & E (original magnification x100). **B** The cytoplasm and membrane of acinar component stained strongly, but lepidic tumor cells stained weakly with L858-specfic antibody (original magnification x100).

The staining distribution included cytoplasm only or cytoplasm together with membrane. Normal tissue adjacent to adenocarcinoma was negative (Figure 2). In the cases with lepidic pattern staining of lepidic tumor cells was either negative or fainter than the tumor cells of other patterns (Figure 3). In our study, 80% of the cases were either negative or positive in 100% of the tumor cells, although the intensity was diverse ranging from + to +++. In only 20% of cases the tumor cells were stained in some areas and completely negative in other areas. Overall, the staining pattern showed characteristics of homogeneity more than heterogeneity.

Based on different scoring systems, the percentage of positive cases was different too. For L858R-specific IHC it was 28% (14/50) on Score A, 16% (8/50) on Score B, and 40% (20/50) on Score C; for E746_A750del-specific IHC it was 20% (10/50) on Score A, 14% (7/50) on Score B, and 24% (12/50) on Score C.

### Concordance analysis of IHC and DNA sequencing

**Figure 4:**
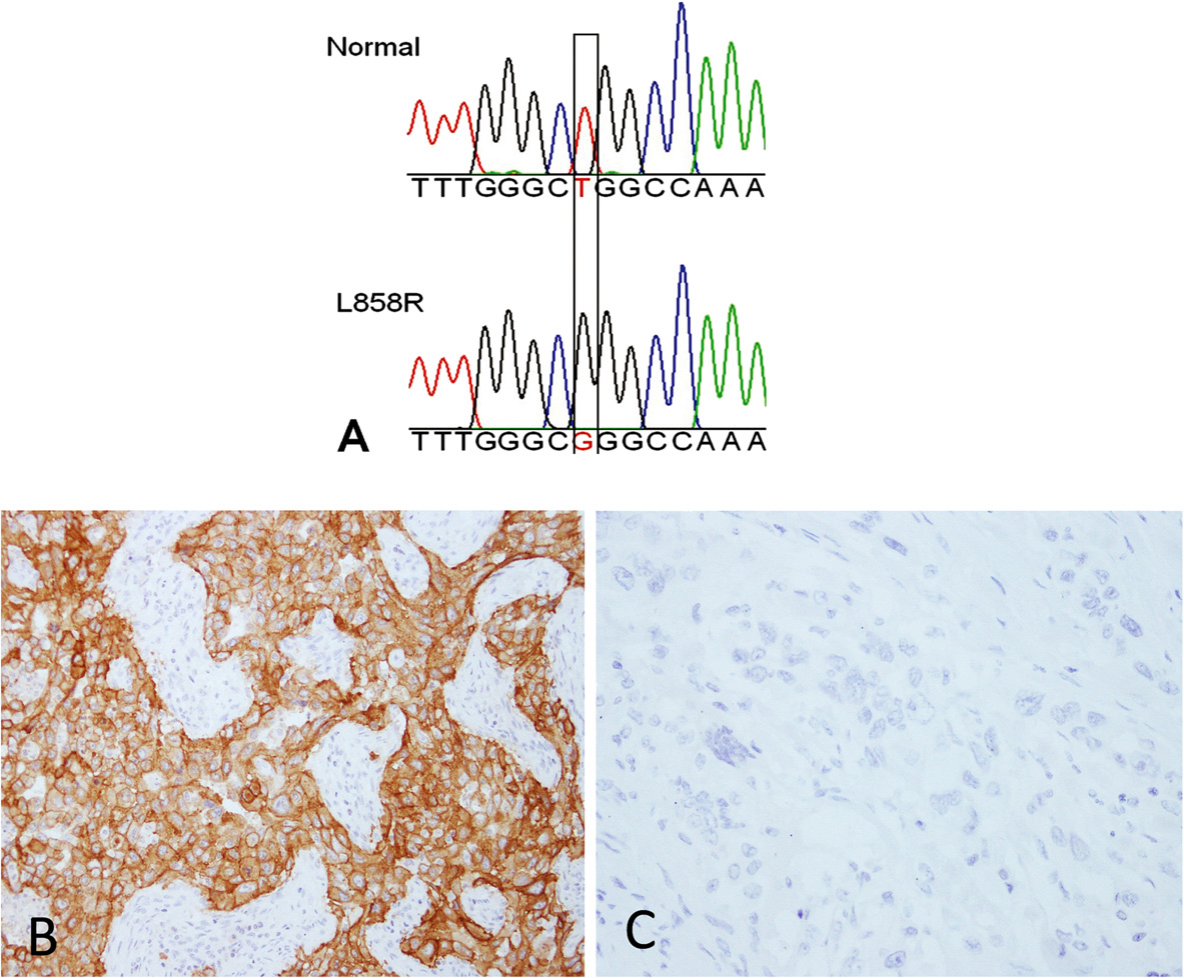
**A case of predominant solid adenocarcinoma with the L858R mutation. A** DNA sequencing of *EGFR* showing normal (upper panel) and L858R mutant (lower panel). The position of the mutation is boxed. The mutant sequence appears to be homozygous with complete absence of normal sequence. This is because of the use of a “clamp” strategy to suppress amplification of the normal sequence, as described in the methods section. **B** Immunohistochemical staining with L858R-specific antibody showing strong positivity (original magnification x200). **C** Immunohistochemical staining with E746-A750 del-specific antibody showing complete negativity (original magnification x200).

**Figure 5:**
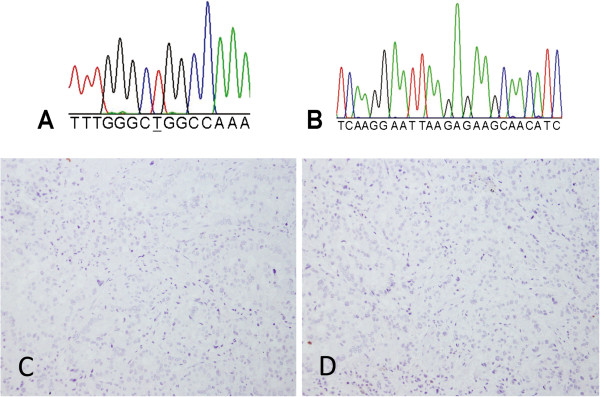
**A case of predominant acinar adenocarcinoma with normal *EGFR. *A** and **B** DNA sequencing shown normal *EGFR* exon 21 and 19, respectively, in the region that is frequently subject to mutation. **C** and **D** Tumor cells were not stained with either L858R-specfic or E746_A750del-specific antibodies, respectively (original magnification x200).

Of the 16 cases with L858R, the L858R-specific IHC was positive in 13 on Score A, 7 on Score B and 11 on Score C (Figure 4). Of 34 cases without L858R the L858R-specific IHC was negative in 33 on Score A, 33 on Score B and 25 on Score C (Figure 5). L858R-specific IHC showed a sensitivity of 81%, a specificity of 97%, a positive predictive value (PPV) of 93%, and a negative predictive value (NPV) of 92% on Score A; a sensitivity of 44%, a specificity of 97%, a PPV of 88%, and a NPV of 79% on Score B; and a sensitivity of 69%, a specificity of 74%, a PPV of 55%, and a NPV of 83% on Score C. Reliability analysis for L858R-specific IHC and DNA sequencing was found to be Cohen’s Kappa = 0.810 and 95% CI (0.701, 0.919) on Score A, Cohen’s Kappa = 0.470 and 95% CI (0.332, 0.608) on Score B, and Cohen’s Kappa = 0.397 and 95% CI (0.261, 0.533) on Score C. The agreement between L858R-specific IHC and DNA sequencing was the best using Score A compared to Score B and C. The difference was significant (P < 0.05) (Table 4).

**Table 4:** Diagnostic power of L858R-specific IHC on score A, B and C

**Scoring system**	**Sensitivity**	**Specificity**	**PPV**	**NPV**	**Cohen’s Kappa**
**A**	82%	97%	93%	92%	0.810 (95% CI: 0.701, 0.919)
**B**	44%	97%	88%	79%	0.470 (95% CI: 0.332, 0.608)
**C**	69%	74%	55%	83%	0.397 (95% CI: 0.261, 0.533)

IHC indicates immunohistochemistry; PPV, positive predictive value; NPV, negative predictive value.

**Figure 6:**
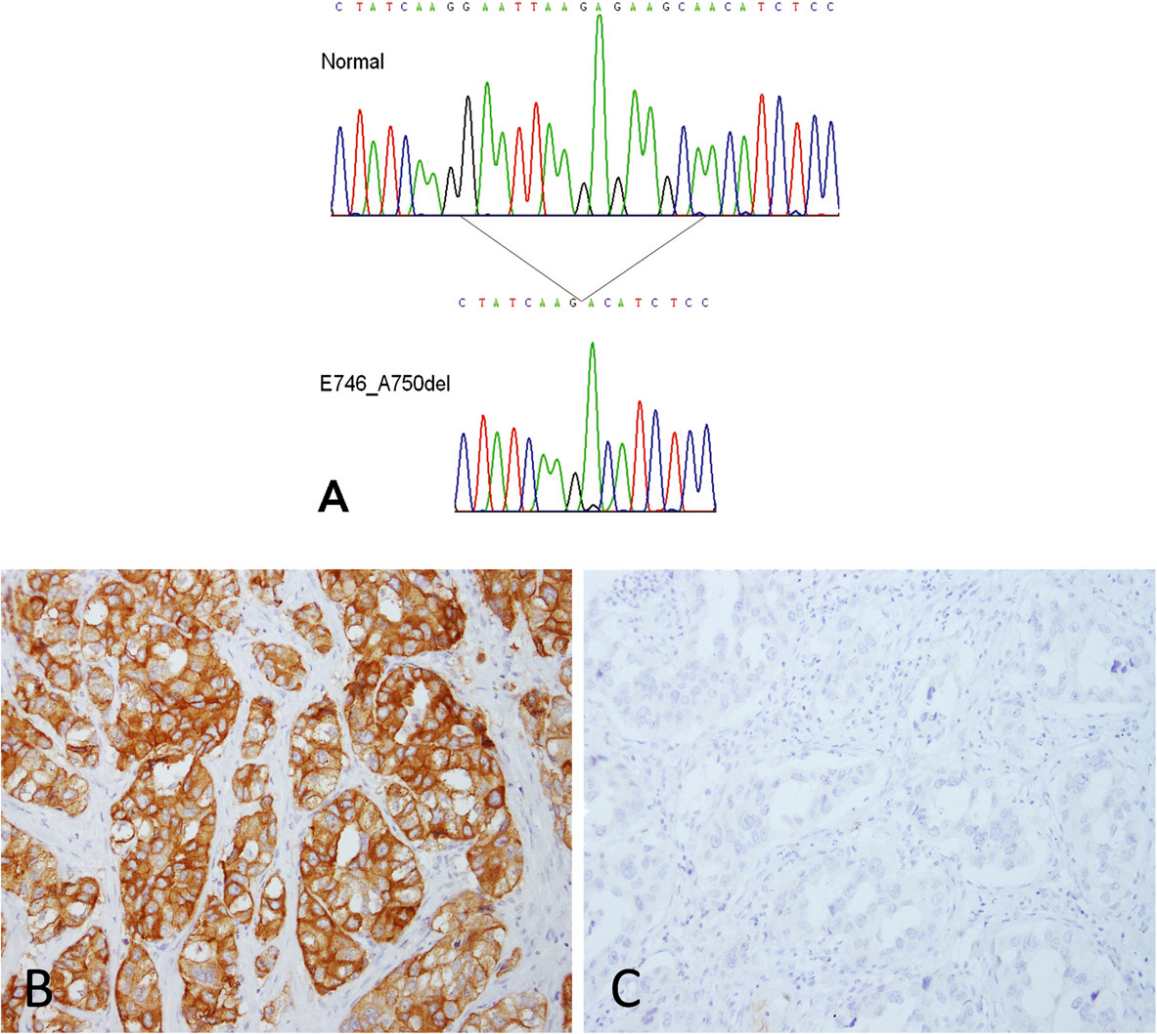
**A case of predominant acinar adenocarcinoma with the exon 19 deletion mutation (E746-A750 del). A** DNA sequencing of *EGFR* showing normal (upper panel) and the E746-A750 del mutant (lower panel). The position of the mutation is indicated. The mutant sequence appears to be homozygous with complete absence of normal sequence. This is because of the use of a “clamp” strategy to suppress amplification of the normal sequence, as described in the methods section. **B** Immunohistochemical staining with E746-A750 del-specific antibody showing strong positivity (original magnification x200). **C** Immunohistochemical staining with L858R-specific antibody showing complete negativity (original magnification x200).

Of 14 cases with the E746_A750del by DNA sequencing E746_A750del-specific IHC was positive in 10 on Score A, 7 on Score B, and 9 on Score C (Figure 6). All of the 3 cases with uncommon types of exon 19 deletion, includingL747_T751del, L747_P753delinsS and L747_T751delinsPT, were negative by E746_A750del-specific IHC regardless of the scoring method.

Including all 17 specimens with an exon 19 deletion detected by DNA sequencing, the E746_A750del-specific IHC found 10 (59%) were positive and 7 (41%) negative on Score A, 7 (41%) were positive and 10 (59%) negative on Score B, 9 (53%) were positive and 8 (47%) negative on Score C. Of the 33 cases without an exon 19 deletion detected by DNA sequencing all were negative by E746_A750del-specific IHC both on Score A and B (Figure 5), while 3 were positive on Score C. As a method to detect deletions in exon 19 despite the exact structure of the deletion, E746_A750del-specific IHC showed a sensitivity of 59%, a specificity of 100%, a PPV of 100%, and a NPV of 82% on Score A; a sensitivity of 41%, a specificity of 100%, a PPV of 100%, and a NPV of 77% on Score B; and a sensitivity of 53%, a specificity of 91%, a PPV of 75%, and a NPV of 79% on Score C. Reliability analysis for E746_A750del-specifiac IHC and DNA sequencing was found to be Cohen’s Kappa = 0.653 and 95% CI (0.521, 0.785) on Score A, Cohen’s Kappa = 0.480 and 95% CI (0.342, 0.618) on Score B, Cohen’s Kappa = 0.472 and 95% CI (0.334, 0.610) on Score C. Similar to L858R-specific IHC the agreement between E746_A750del-specific IHC and DNA sequencing was the best using Score A compared to Score B and C, but the difference was not significant (P > 0.05) (Table 5).

**Table 5:** Diagnostic power of E746_A750del-specific IHC on score A, B and C

**Scoring system**	**Sensitivity**	**Specificity**	**PPV**	**NPV**	**Cohen’s Kappa**
**A**	59%	100%	100%	82%	0.653 (95% CI: 0.521, 0.785)
**B**	41%	100%	100%	77%	0.480 (95% CI: 0.342, 0.618)
**C**	53%	91%	75%	79%	0.472 (95% CI: 0.334, 0.610)

IHC indicates immunohistochemistry; PPV, positive predictive value; NPV, negative predictive value.

## Discussion

The studies that established the relationship between mutations in the EGFR gene and response to the small molecule EGFR TKIs gefitinib and erlotinib were done using analysis of DNA extracted from the tumor [18]. The recent availability of antibodies that are specific for the mutations most clearly associated with response to EGFR TKIs, L858R and E746_A750del, create the opportunity to exploit an alternative method to evaluate NSCLC for EGFR mutations to aid decisions with regard to EGFR TKI therapy [11].

IHC has the advantage of being a method that is routinely applied in solid tumor diagnosis in pathology. Also, it can be used on specimens that are not optimal for DNA analysis such as small tissue samples or individual cells obtained from body fluids, bronchial washings, and fine needle aspirates. Although some studies have shown that EGFR gene sequencing could be successfully applied to cytological specimens [19], it is still a problem to get enough DNA for sequencing from such samples in routine practice. Thus, the development of antibodies that specifically detect mutant EGFR protein by IHC could be a valuable addition to the current methods used to diagnose and predict response to treatment of lung cancer.

In 2009, Yu et al. [7] reported generating two mAbs from New Zealand rabbits, one against the E746_A750del and the other against the L858R point mutation, and evaluated them by Western blotting, immunofluorescence and IHC. They tested these antibodies in a series of cell lines and in tumor tissues from patients with primary NSCLC, with known and unknown EGFR mutations, comparing the IHC results with DNA sequencing. They found that IHC with these mutation-specific antibodies showed a sensitivity of 92% and a specificity of 99%. Recently, several studies examined the presence of EGFR mutations in NSCLC by IHC using the same two antibodies and the reported sensitivity ranged from 24% to 100% and specificity ranged from 77% to 100% [8-15]. IHC is known to sometimes suffer from high inter-laboratory variability in assay performance, and high inter-observer variability in assay interpretation. These drawbacks may explain the variability in results of the studies described above. There is still much work to be done before IHC can be considered an adequate substitute for direct analysis of mutations in the EGFR gene in NSCLC.

In our study we found that slides treated by EDTA (pH 8.0) showed the best histological pictures with strongly specific staining and minimal background. As a result when pathologists reviewed these slides the intra- and inter-observer agreement was better than those treated by sodium citrate (pH 6.0) and EDTA (pH 9.0). The difference was statistically significant (P < 0.05). In conclusion, EDTA (pH 8.0) is the preferred buffer for antigen retrieval for IHC using EGFR mutation specific antibodies.

Scoring is the final step involved in the IHC protocol, but is not the least one, because the scoring system plays a critical role in obtaining a reliable result. In our study, we compared three scoring systems that have been used by other investigators, using DNA sequencing as the gold standard [10,13,15]. For L858R-specific IHC the agreement with DNA sequencing using Score A was superior to Score B and C. The difference was statistically significant. For E746_A750del-specific IHC the agreement with DNA sequencing was good for Score A, which was superior to Score B and C, but the difference was not statistically significant. In conclusion our study showed that Score A is the most appropriate way to interpret the EGFR mutation-specific IHC.

Based on Score A the specificity of EGFR mutation-specific IHC was very high, 100% for exon 19 deletions and 97% for L858R, while sensitivity was lower, 81% for L858R and 59% for exon 19 deletions. In another words, mutation-specific IHC demonstrated extremely high specificities, but much lower sensitivity. The low sensitivity of the exon 19 del IHC is mostly due to the presence of several exon 19 deletions other than the most common E746_A750del, which is the target of the exon 19 del antibodies. We conclude, based on our work, that NSLC cases positive by IHC could be selected as candidates for EGFR-TKI, while negative cases should be referred for further testing by DNA analysis.

In our study the majority of cases were either negative or positive in 100% of the tumor cells. The staining pattern showed characteristics of homogeneity more than heterogeneity. Consequently, we expect that evaluation of the mutation status by IHC should be reliable using small biopsy specimens or tissue microarray.

Our study also showed that all of the normal alveolar epithelial cells were completely negative and the intensity of mutation-specific immunostaining was much fainter in tumor cells with a lepidic pattern comparing to other patterns. This demonstrates that the EGFR mutations are tumor-specific, and likely an initiating event in lung cancer tumorigenesis.

## Conclusions

Immunohistochemistry using mutation-specific mAbs is demonstrated to be a reliable test for detecting EGFR mutations in adenocarcinoma of the lung in our study. The diagnostic power of EGFR mutation-specific IHC is influenced by the antigen retrieval and scoring methods. Based on our study EDTA (pH 8.0) is better than sodium citrate (pH 6.0) and EDTA (pH 9.0) as the antigen retrieval buffer. A practical and reliable scoring method, i.e. positive is interpreted as moderate to strong staining of membrane and/or cytoplasm in >10% tumor cells, is recommended. However its final validation depends on strict quality control of the whole protocol, including antibody manufacture, IHC method, scoring system, criteria for interpretation, and the proper way to integrate with molecular methods, etc.

The specificity of EGFR mutation-specific IHC was very high, 100% for exon 19 deletions and 97% for L858R, while sensitivity was relatively lower, 81% for L858R and 59% for Exon 19 deletions. Considering the use of IHC has the advantage of being a method routinely applied in solid tumor diagnosis in pathology, EGFR mutation-specific IHC could be used as a prescreening method for selecting EGFR-TKI candidates. The positive cases by IHC could be selected as candidates for EGFR-TKI, while negative cases should be referred for DNA analysis. Additionally, as the staining pattern showed characteristics of homogeneity more than heterogeneity, it should be reliable to evaluate the mutation status of biopsy specimens or tissue microarray using IHC. Furthermore, it may be possible to use IHC as a substitute when the quantity of the sample DNA is not sufficient for molecular methods, e.g., small tissue samples or individual cells obtained from body fluids, bronchial washings, and fine needle aspirates etc.

## Consent

Written informed consent was obtained from the patient for publication of this report and any accompanying images.

## Abbreviations

ARMS: Amplification refractory mutation system; CI: Confidence interval; CLMP1: Clamps for exons 19; CLMP2: Clamps for exons 21; EGFR: Epidermal growth factor; FFPE: Formalin-fixed paraffin- embedded; IHC: Immunohistochemistry; LNA: Locked nucleic acid; NSCL: Non-small cell lung cancer; NPV: Negative predictive value; PPV: Positive predictive value; TK: Tyrosine kinase; TKI: Tyrosine kinase inhibitor.

## Competing interests

The authors declare that we do not have any financial competing interests.

## Authors’ contributions

XY participated in the design of the study and drafted the manuscript. BY carried out the immunoassays and collected patient’s clinic data. LN and LTS carried out the molecular genetic studies. RPG carried out the molecular genetic studies, and helped to draft the manuscript. XH contributed to the design of the study, helped to draft the manuscript, and participated in coordination. NL, ZJ and DY participated in the review of the histologic slides. NL carried out the molecular genetic studies as well. LT conceived of the study, participated in its design and coordination, and helped to draft the manuscript. All authors read and approved the final manuscript.

## Authors’ information

Yan Xiong, MD, Associated Professor in Department of Pathology, Peking University First Hospital, Beijing, China. Ting Li, MD, Full professor and Chair in Department of Pathology, Peking University First Hospital, Beijing, China.

## Supplementary Material

Additional file 1 Protocol for EGFR mutation-specific immunohistochemistry. Click here for file
