# Supplementary material for: Immunohistochemical detection of mutations in the epidermal growth factor receptor gene in lung adenocarcinomas using mutation-specific antibodies
Source: Diagn Pathol. 2013 Feb 18;8:27. doi: 10.1186/1746-1596-8-27 (PMC3635899; doi:10.1186/1746-1596-8-27)
Supplement: Additional file 1 — Protocol for EGFR mutation-specific immunohistochemistry. [file 1746-1596-8-27-S1.docx]

**Supplementary material**

**Protocol for EGFR mutation-specific immunohistochemistry**

1. Before immunostaining, the slides are heated (56 ˚C) for 3 hours in a drying oven.
2. Slides are deparaffinized (xylene), washed with alcohol (100% and 95%), and rehydrated in deionized water.
3. Antigen retrieval is performed as follows
4. Retrieval solution (EDTA pH8.0) is prepared in advance: To prepare 1 L adding 0.372 g EDTA (C10H14N2O8Na2•2H2O) to 1 L dH2O and adjusting pH to 8.0
5. Set pressure cooker to 103 kPa which ought to achieve a temperature of 120 °C at full pressure. Fill the pressure cooker with enough retrieval solution to cover slides. Bring contents to near boiling point, place racked slides into retrieval solution, seal the pressure cooker, and again bring the solution to a boil for 2 minutes. Then allow slides to cool to room temperature before rinsing with Trisbuffered saline wash buffer (Dako).
6. Endogenous peroxidase activity is blocked by incubating the slides for 5 minutes in 0.03% hydrogen peroxide (EnVision/HRP, Dako).
7. After rinsing in wash buffer, the sections are incubated for 1 hour at room temperature with EGF Receptor (E746-A750del Specific) (6B6) XP™ Rabbit mAb (Cell Signaling, Danvers, MA) and EGF Receptor (L858R Mutant Specific)(43B2) Rabbit mAb (Cell Signaling, Danvers, MA), both of which are diluted to 1:100 in Tris-HCl buffer antibody diluent (Dako).
8. Slides are rinsed in wash buffer, then incubated for 30 minutes with peroxidase-labeled polymer conjugated to goat anti-rabbit immunoglobulins (EnVision/HRP, Dako).
9. The chromogenic reaction is carried out with 3, 3’-diaminobenzidine chromogen solution for 5 minutes, resulting in the expected browncolored signal.
10. After rinsing with deionized water, the slides are counterstained with hematoxylin, dehydrated, mounted with toluene-based mounting medium (Thermo Scientific Richard-Allan) and coverslipped.
